# Supplementary material for: Deep Neural Networks for Dental Implant System Classification
Source: Biomolecules. 2020 Jul 1;10(7):984. doi: 10.3390/biom10070984 (PMC7407934; doi:10.3390/biom10070984)

Deep Neural Networks for Dental Implant System Classification

Shintaro Sukegawa [1,2,*], Kazumasa Yoshii [3], Takeshi Hara [3], Katsusuke Yamashita [4], Keisuke Nakano [2], Norio Yamamoto [5], Hitoshi Nagatsuka [2] and Yoshihiko Furuki [1]

[1] Department of Oral and Maxillofacial Surgery, Kagawa Prefectural Central Hospital, 1-2-1, Asahi-machi, Takamatsu, Kagawa, 760-8557, Japan; gouwan19@gmail.com
[2] Department of Oral Pathology and Medicine, Okayama University Graduate School of Medicine, Dentistry and Pharmaceutical Sciences, Okayama, 700-8558, Japan
[3] Department of Electrical, Electronic and Computer Engineering, Faculty of Engineering, Gifu University, 1-1 Yanagido, Gifu, Gifu, 501-1193, Japan
[4] Polytechnic Center Kagawa, 2-4-3, Hananomiya-cho, Takamatsu, Kagawa 761-8063, Japan
[5] Department of Orthopaedic Surgery, Kagawa Prefectural Central Hospital, Takamatsu, Kagawa, 760-8557, Japan

* Correspondence: gouwan19@gmail.com; Tel.: +81 87 811 3333, Fax: +81 87 835 8363

Appendix

Figure S1. Mean ROC curves of each CNN models for 11 types of dental implant classification

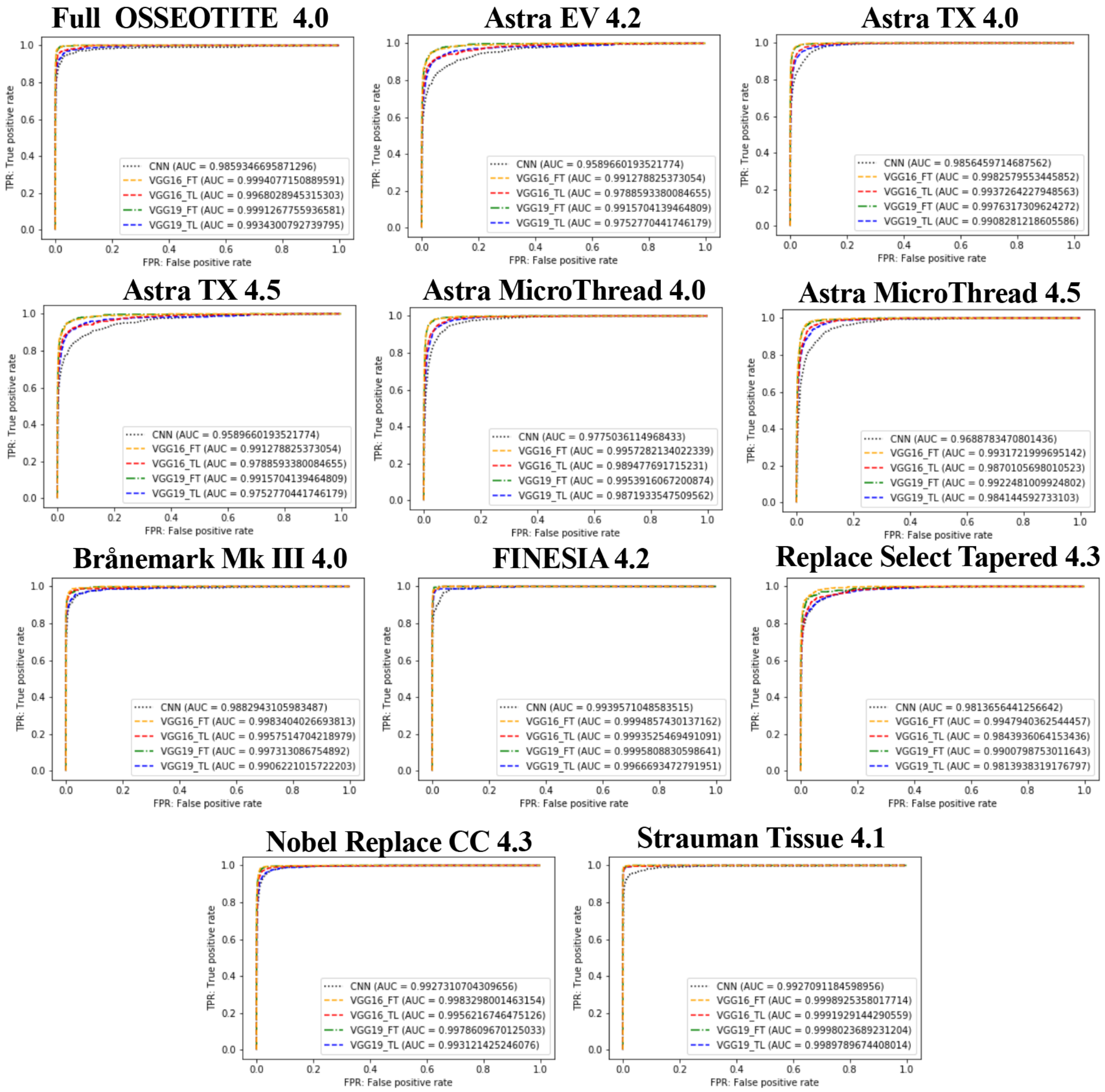

Abbreviation in Figure S1
CNN; Basic CNN model with three convolutional layers (basic CNN)
VGG16_FT; Fine-tuning VGG16 model with pre-trained weights (VGG16 fine tuning)
VGG16_TL; Transfer-learning VGG16 model with pre-trained weights (VGG16 transfer)
VGG19_FT; Fine-tuning VGG19 model with pre-trained weights (VGG19 fine tuning)
VGG19_TL; Transfer-learning VGG19 model with pre-trained weights (VGG19transfer)

Figure S2. Visualization of Model Classification

Figure S2 shows ten representative images of 11 types of dental implants classified using each CNN model, visualized using Grad-CAM.

Full OSSEOTITE 4.0

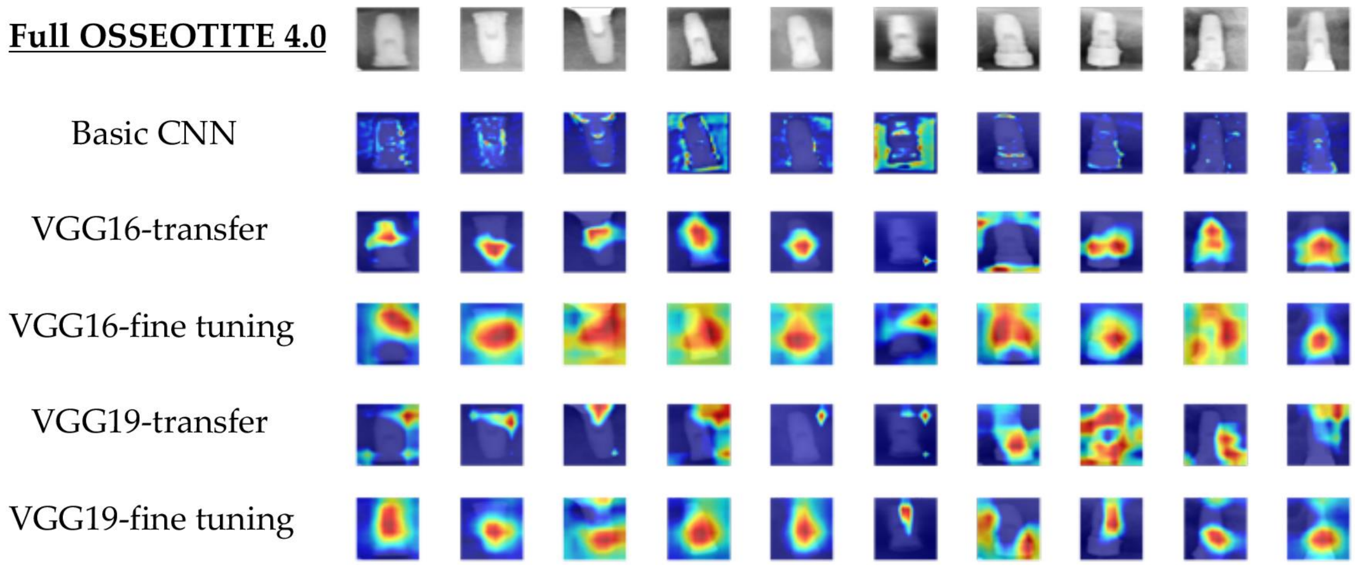

Astra EV 4.2

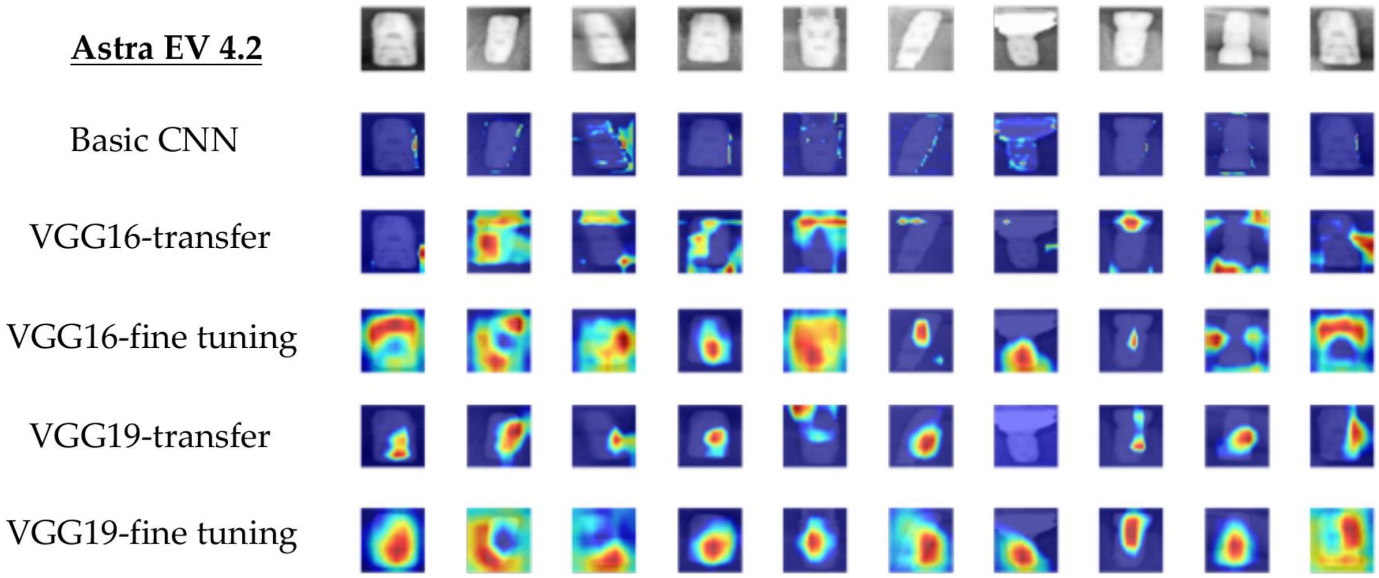

Astra TX 4.0

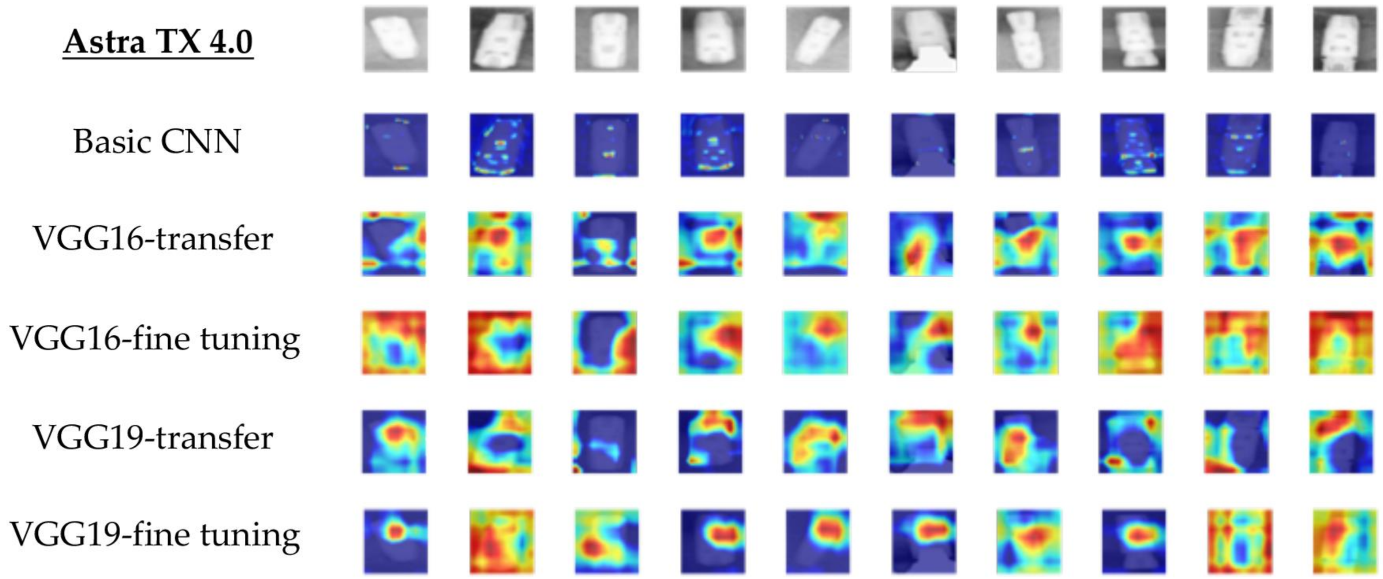

Astra TX 4.5

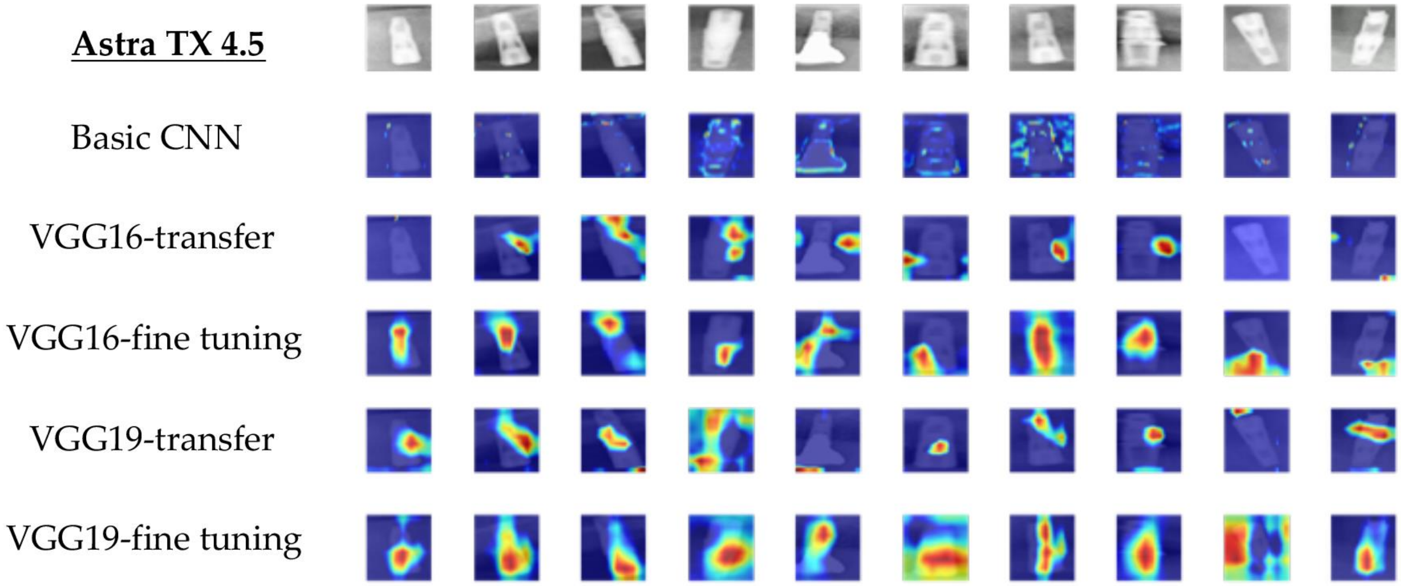

Astra MicroThread 4.0

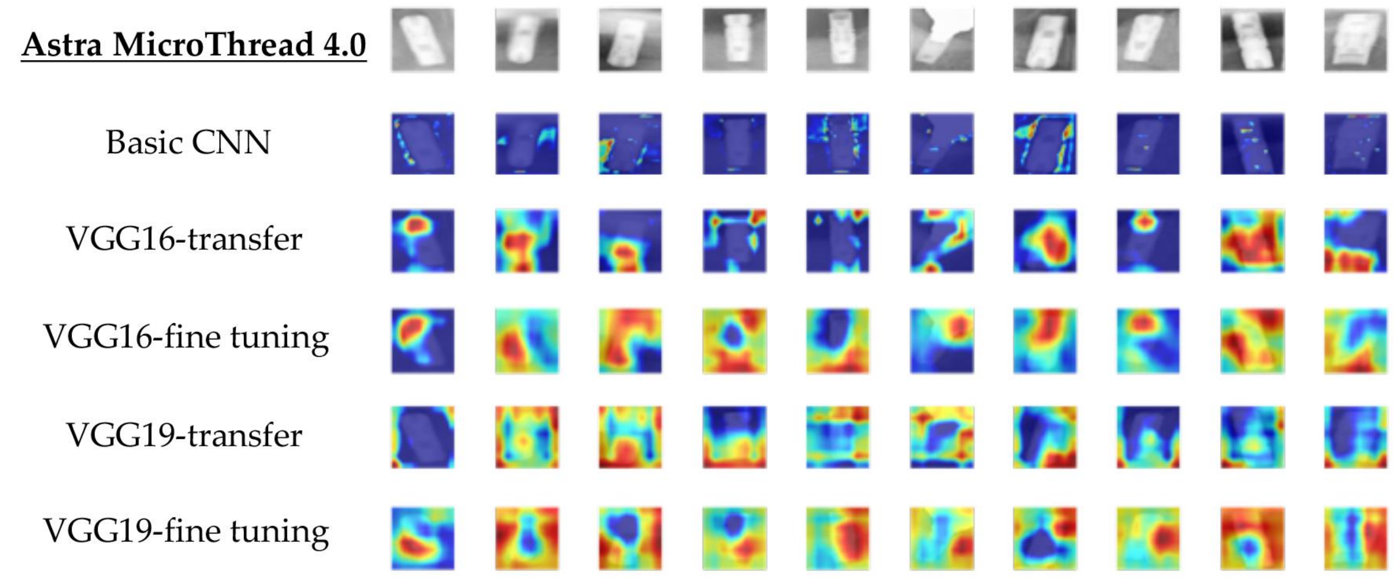

Astra MicroThread 4.5

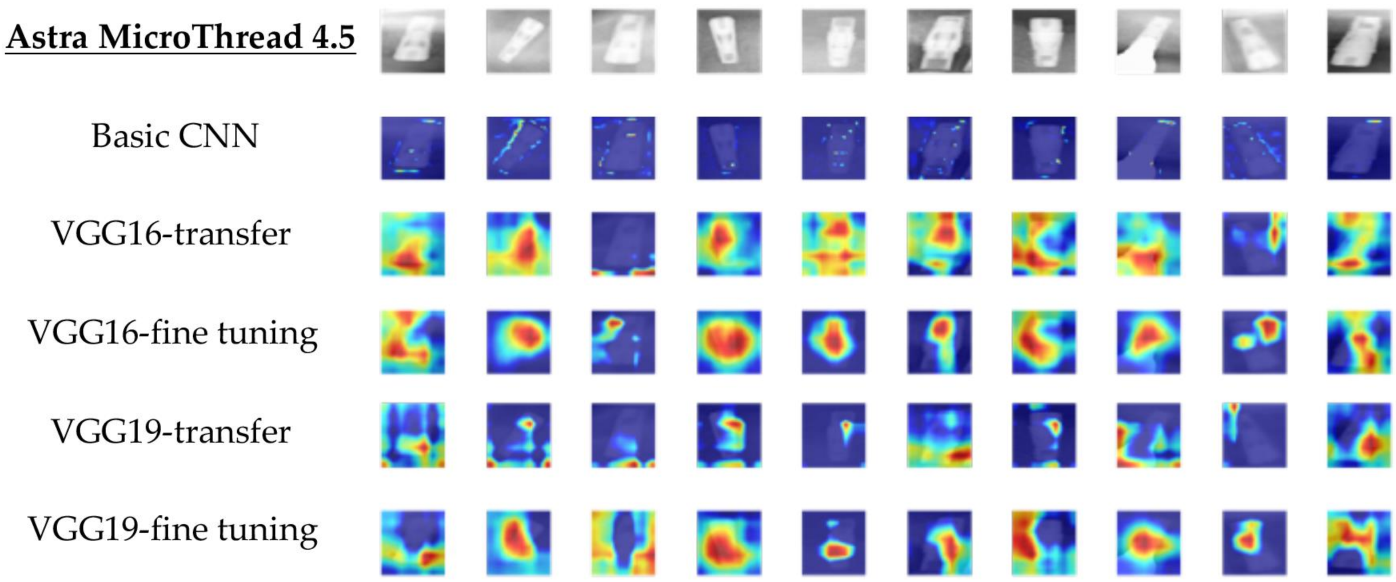

Brånemark Mk III 4.0

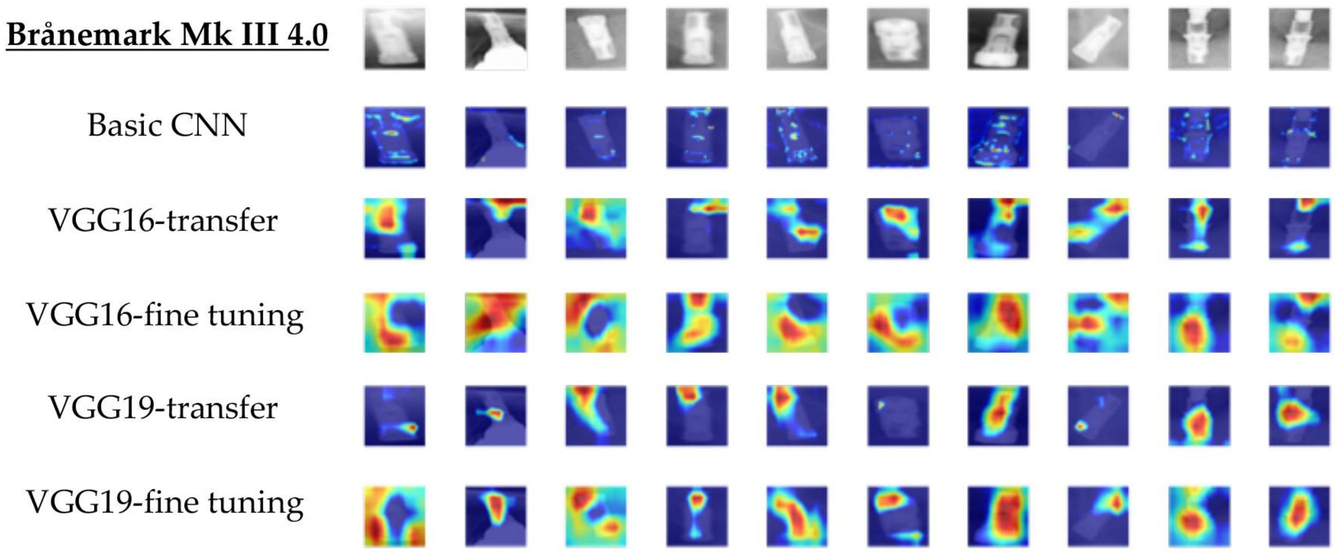

FINESIA 4.2

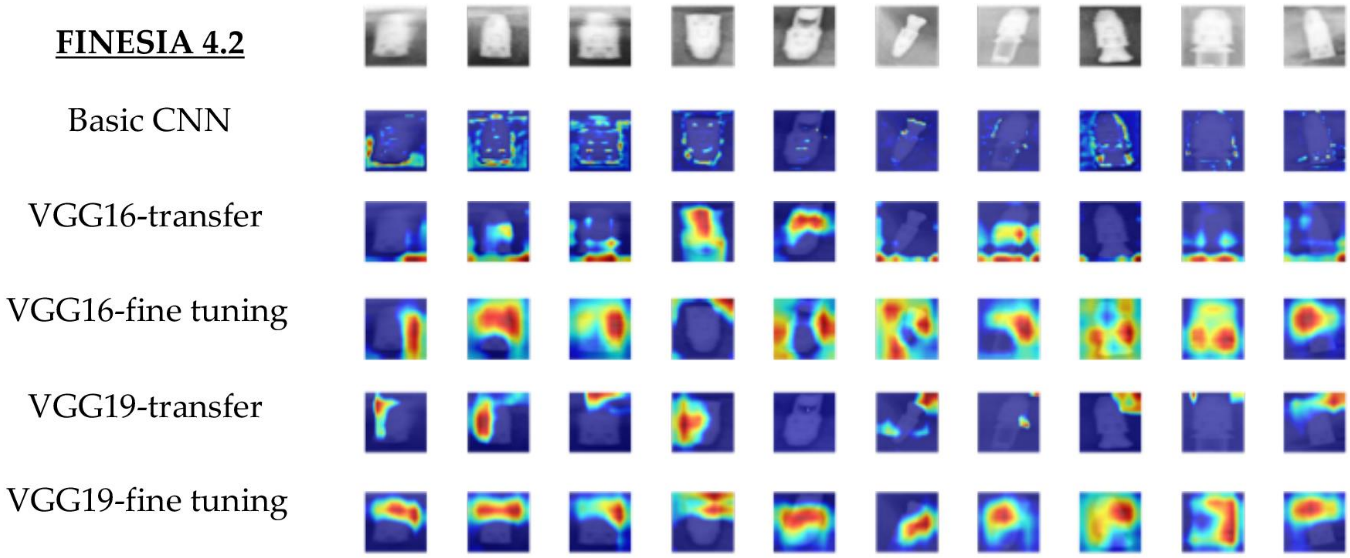

Replace Select Tapered 4.3

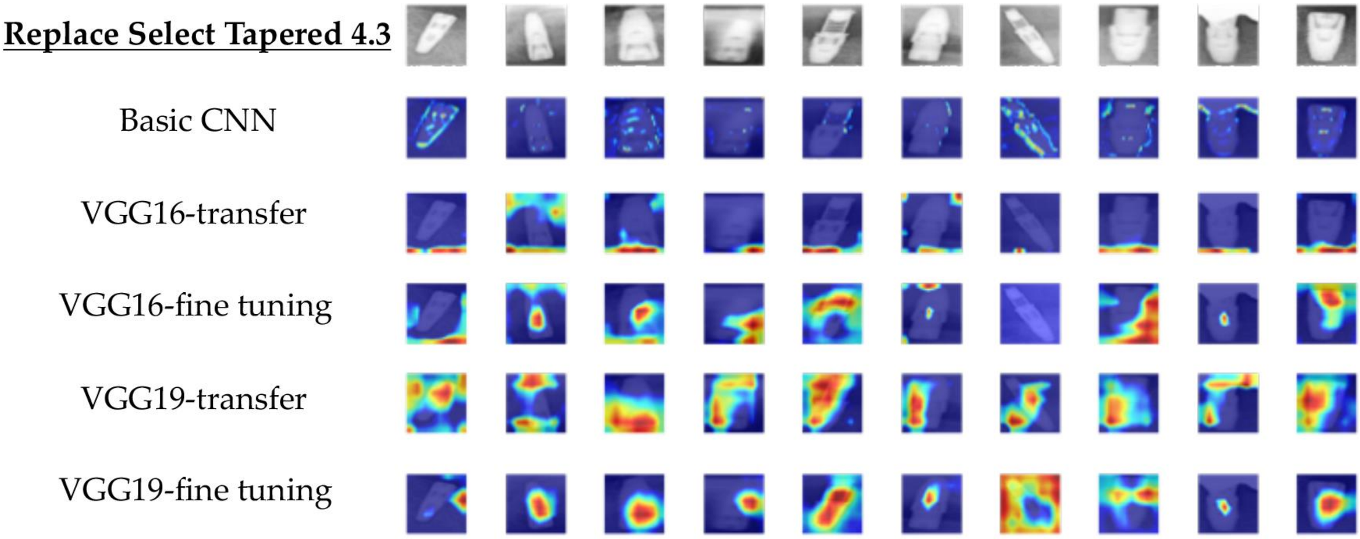

Nobel Replace CC 4.3

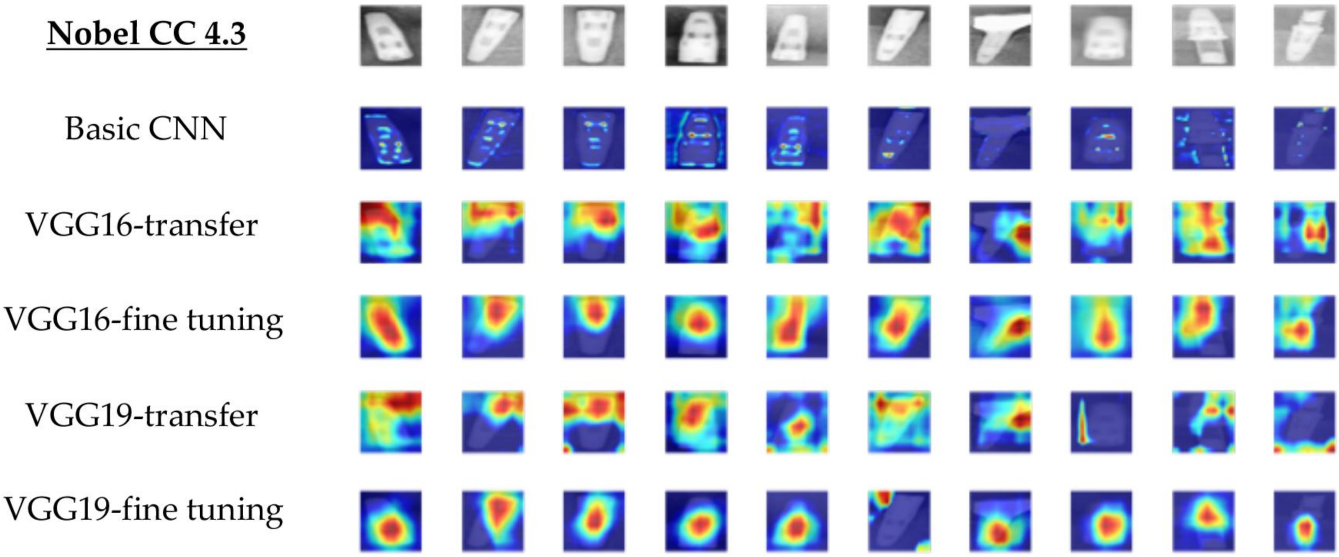

Strauman Tissue 4.1

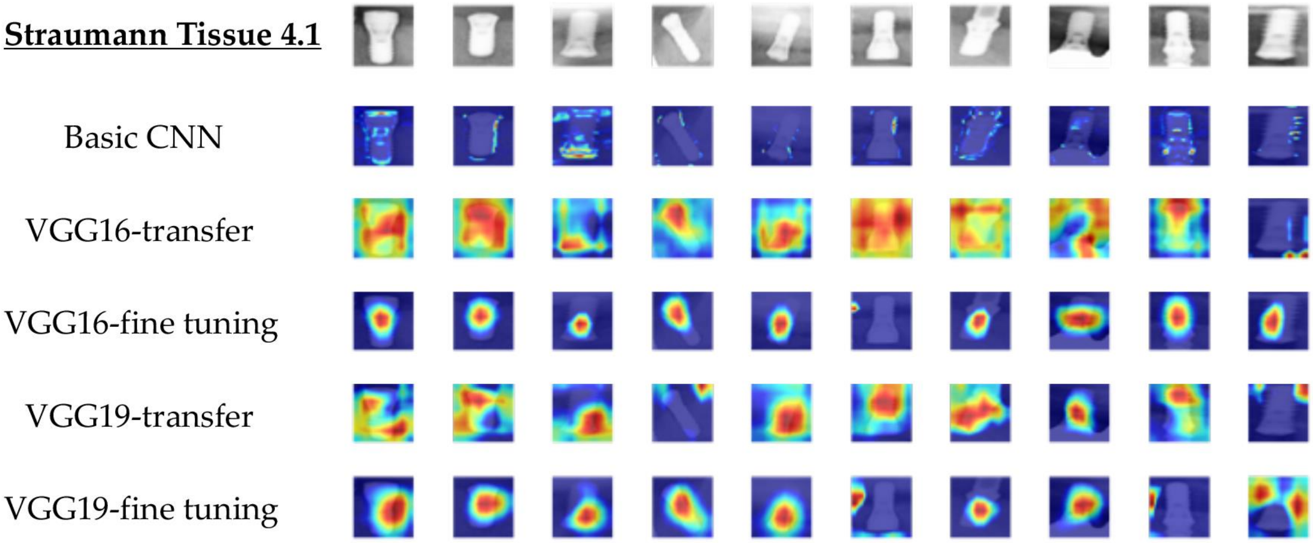

Supplement: Supplementary file 1 [file biomolecules-10-00984-s001.pdf]
